# Rare Cause of Congenital Unilateral Facial Palsy: High-Resolution Computed Tomography (HRCT) Findings of Isolated Facial Canal Hypoplasia and Aplasia

**DOI:** 10.7759/cureus.115085

**Published:** 2026-08-24

**Authors:** Keerthana Suresh, Umashankar Baskar, Venkata Sai, S Rohith, Priya Mani

**Affiliations:** 1 Radiodiagnosis, Sri Ramachandra Institute of Higher Education and Research, Chennai, IND; 2 Radiology, Sri Ramachandra Institute of Higher Education and Research, Chennai, IND

**Keywords:** congenital facial nerve anomaly, congenital facial nerve palsy, congenital unilateral facial palsy, developmental facial nerve anomaly, facial canal aplasia, facial canal hypoplasia, facial nerve canal agenesis, fallopian canal anomaly, hrct temporal bone, isolated facial nerve anomaly

## Abstract

Congenital facial nerve palsy (CFNP) is an uncommon medical condition conventionally grouped into acquired (traumatic) and developmental (isolated or syndromic) categories depending on the cause. Distinction between the two etiological mechanisms is mandatory for determining further prognosis and selecting the treatment method. High-resolution computed tomography (HRCT) of the temporal bone currently remains the gold-standard diagnostic technique used to examine the complex osseous architecture of the Fallopian canal and reveal congenital abnormalities.

In this report, we describe the case of a four-year-old girl who had right-sided congenital facial nerve palsy in the form of asymmetric facies and deviation of the angle of the mouth to the left side since birth. Her perinatal history was unremarkable, with no evidence of birth trauma or prolonged labor. To rule out anatomical anomalies, HRCT of the temporal bones was performed. The study revealed a selectively hypoplastic labyrinthine segment of the right facial canal, progressing to severe hypoplasia and complete aplasia of the tympanic and mastoid segments. Notably, the adjacent middle ear ossicles, bony labyrinth, and contralateral temporal bone structures were entirely normal, ruling out broader syndromic association.

Isolated congenital agenesis and hypoplasia of the facial nerve canal, without associated external, middle, or inner ear anomalies, is an extremely rare embryological aberration causing CFNP. This case clearly illustrates the key role of HRCT in establishing a definitive diagnosis of developmental facial palsy, thereby preventing unnecessary surgical decompressions and guiding parental counseling.

## Introduction

Congenital facial nerve palsy (CFNP) is defined as clinical facial paralysis present at birth or noted immediately in the neonatal period. This is a rather rare entity with an estimated incidence of about 2 per 1,000 live births [1]. The etiology of CFNP can be categorized into two types: acquired and developmental [1]. Acquired CFNP, which accounts for the vast majority of cases (approximately 78% to 91%), is typically secondary to intrauterine trauma, forceps delivery, or prolonged labor, and generally carries an excellent prognosis for spontaneous recovery [2].

On the other hand, developmental CFNP is caused by genetic mutations, teratogenic agents, or embryological anomalies. It is often syndromic, presenting as part of a broader spectrum of congenital anomalies such as Moebius syndrome, Goldenhar syndrome, or hemifacial microsomia [3]. In general, developmental anomalies are classically associated with malformation of the external ear (microtia, atresia), middle ear (ossicles), or inner ear (labyrinthine dysplasia), as these organs share similar embryology.

The facial nerve possesses the most complex and tortuous anatomical osseous course compared to any other cranial nerve, traversing the temporal bone through the Fallopian canal. This makes it susceptible to developmental errors. Computed tomography (CT) allows an indirect view of the nerve and is highly useful to demonstrate the intraosseous segments of cranial nerves and their pathologic changes [4]. High-resolution computed tomography (HRCT) of the temporal bone is the imaging modality of choice for delineating the anatomy of the osseous fallopian canal, allowing for precise morphological evaluation [4].

This report describes a unique case of a four-year-old female presenting with isolated right facial nerve palsy secondary to severe hypoplasia and aplasia of the right facial nerve canal.

## Case presentation

A four-year-old female child presented to the outpatient department for the evaluation of facial asymmetry that had been present since birth. The parents reported a persistent deviation of the angle of the mouth to the left side, an inability to fully close the right eye, and asymmetric crying facies. The clinical examination was consistent with a right-sided lower motor neuron facial nerve palsy. There were no obvious dysmorphic facial features, microtia, or external ear deformities to suggest a syndromic association.

A detailed perinatal history was obtained. The antenatal, natal, and postnatal periods were uneventful. The child was born at term via spontaneous vaginal delivery without the use of instrumentation such as forceps or vacuum extraction. There was no history of prolonged labor, neonatal intensive care unit (NICU) admission, neonatal fever, or incessant crying. The child’s developmental milestones, including speech and motor skills, were appropriate for her age, and she was vaccinated according to the national immunization schedule.

To evaluate the osseous structures of the temporal bone and to trace the precise course of the facial nerve, a plain HRCT of the temporal bones was performed utilizing serial thin coronal and axial sections.

Imaging findings

**Figure 1:**
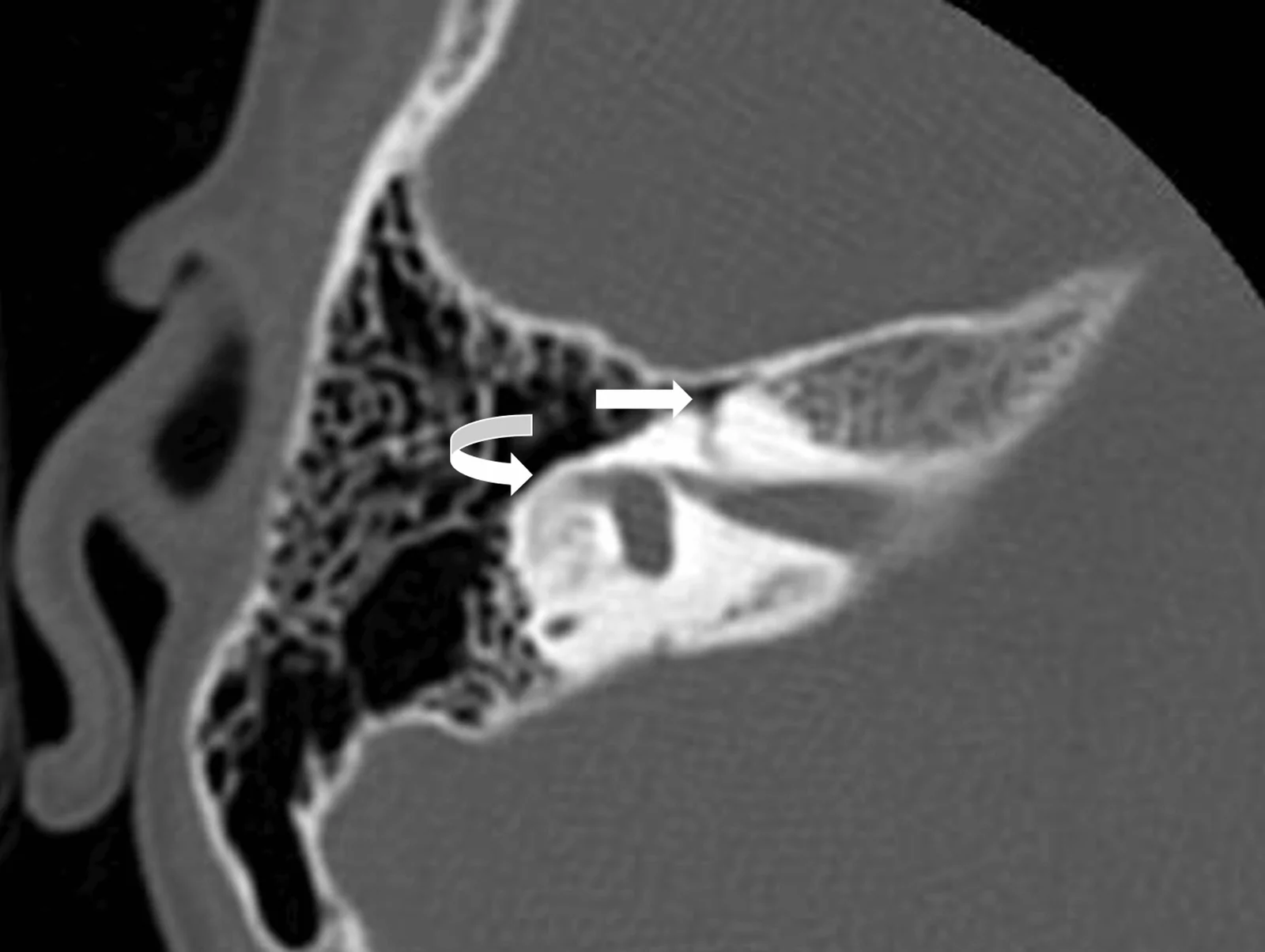
High-resolution computed tomography (HRCT) of the right temporal bone: axial section The images demonstrate a hypoplastic labyrinthine segment and severe hypoplasia of the tympanic segment of the facial nerve canal. Straight arrow: hypoplastic first genu and labyrinthine part of the facial nerve canal; curved arrow: severe hypoplasia of the tympanic part of the facial nerve canal

**Figure 2:**
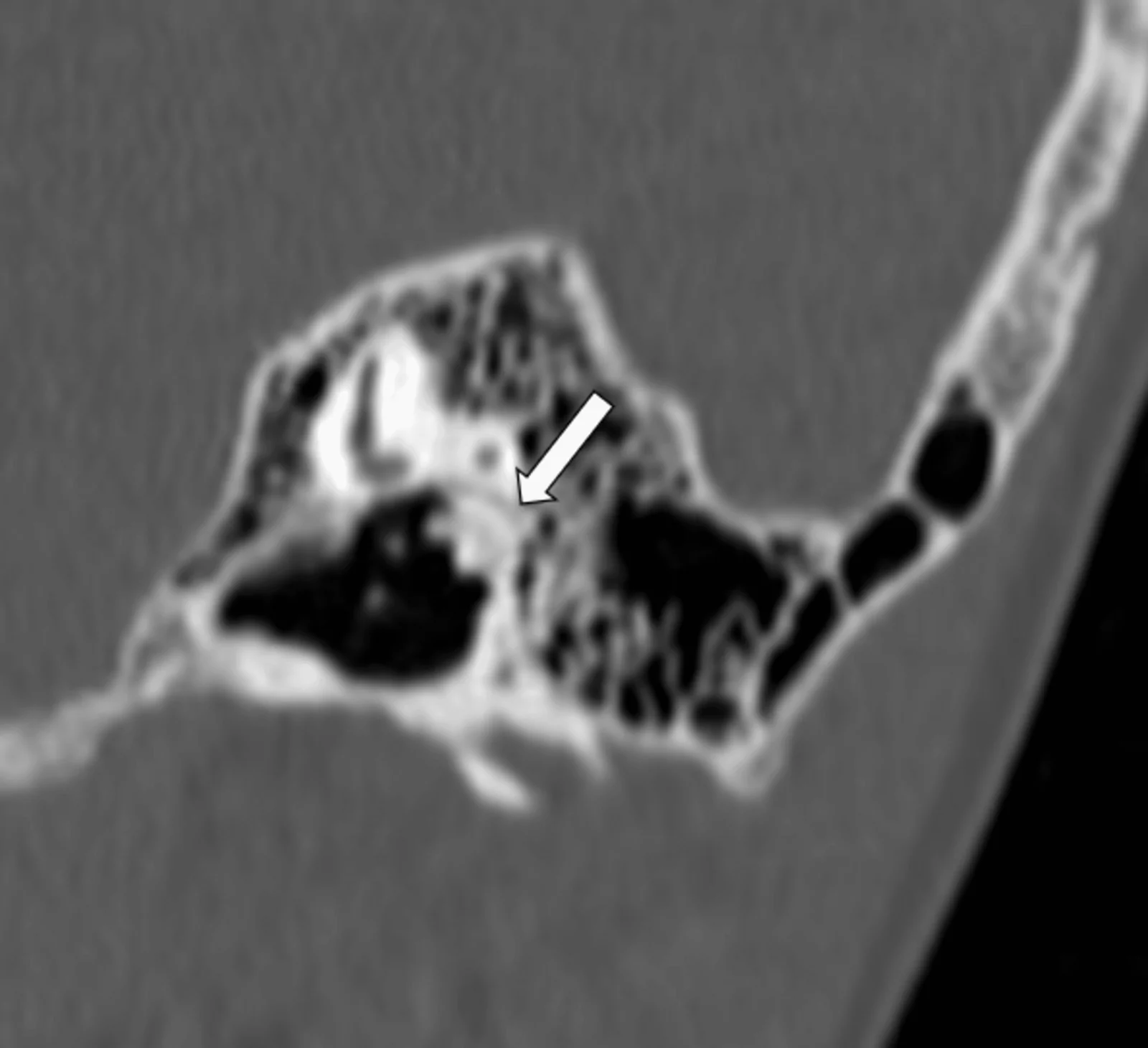
High-resolution computed tomography (HRCT) of the right temporal bone - oblique sagittal section The images demonstrate severe hypoplasia of the tympanic and aplasia of the mastoid segments of the facial canal. Straight arrow: severe hypoplasia of the tympanic part of the facial nerve canal Note that there is complete aplasia of the mastoid part of the facial nerve canal.

Imaging of the temporal bones was performed using a 64-slice GE Revolution EVO CT scanner (GE Healthcare, Chicago, IL, US) with a slice thickness of 0.6 mm, a tube voltage of 140 kVp, and a tube current of 150 mA. A bone reconstruction algorithm was used. The HRCT study demonstrated significant architectural abnormalities strictly isolated to the course of the right facial nerve. The labyrinthine segment of the right facial canal, up to the geniculate ganglion, appeared hypoplastic. Distal to this, the tympanic segment and the descending mastoid segments of the right facial canal demonstrated severe hypoplasia and areas of complete aplasia (Figures 1, 2).

Remarkably, the surrounding regional anatomy of the external, middle, and inner ear on the right side was entirely preserved. The bony and cartilaginous external auditory canal appeared normal without soft tissue occlusion. The middle ear cavity (epitympanum, mesotympanum, and hypotympanum) was normal. The ossicular chain (malleus, incus, and stapes) and their respective malleoincudal and incudostapedial articulations appeared intact and normal. The tegmen tympani, scutum, and tympanic annulus showed no defects. The medial wall structures, including the cochlear promontory, lateral semicircular canal, and the round and oval windows, were normal with no evidence of lytic or sclerotic foci. The bony labyrinth (cochlea, vestibule) and the internal auditory meatus were normal in size. The jugular bulb and carotid canal were also unremarkable.

**Figure 3:**
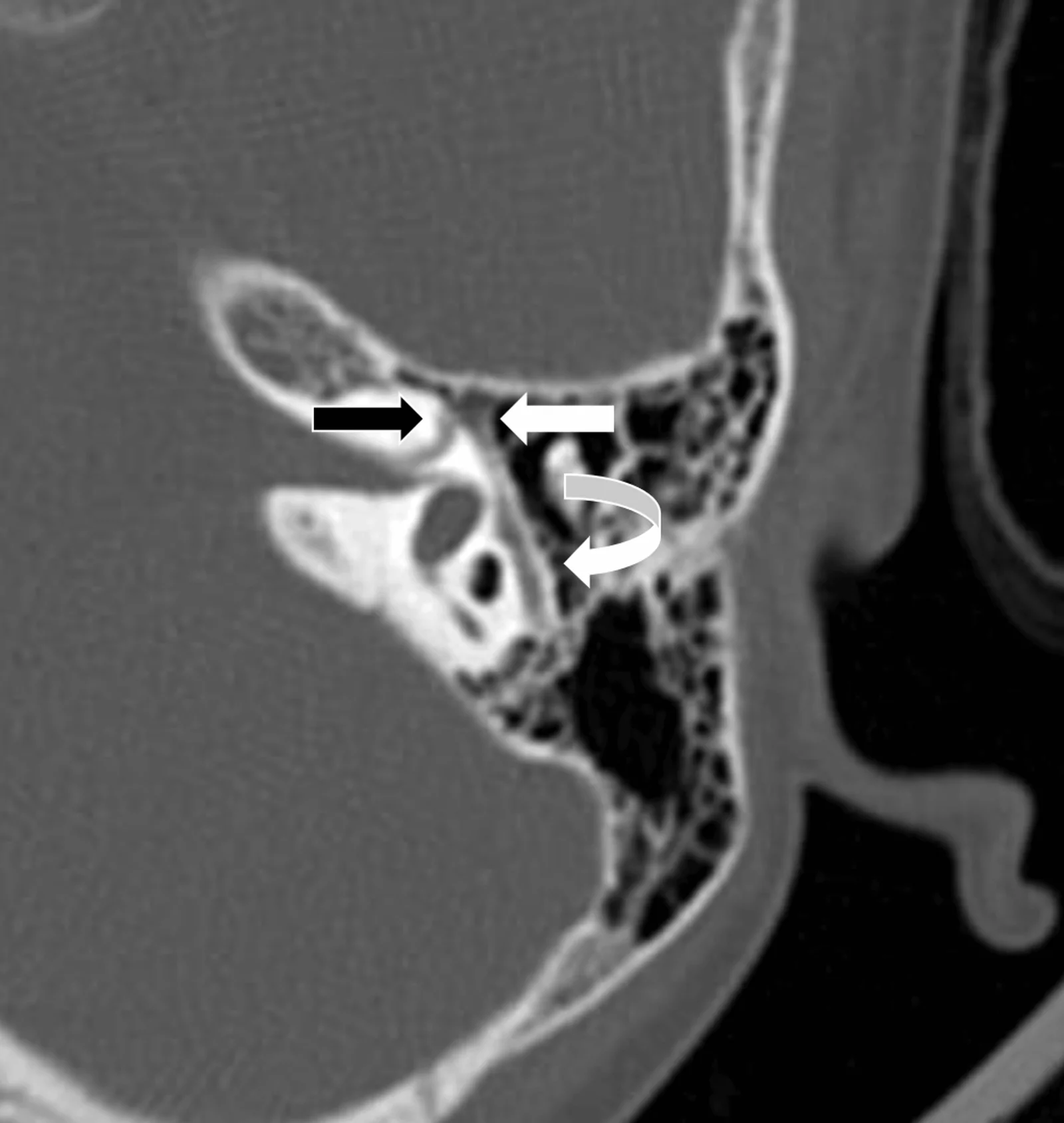
High-resolution computed tomography (HRCT) of the left temporal bone - axial section The images demonstrate a normal, well-defined bony facial canal for comparison. Black straight arrow: labyrinthine part of the facial nerve canal; white straight arrow: first genu of the facial nerve canal; curved arrow: tympanic part of the facial nerve canal

**Figure 4:**
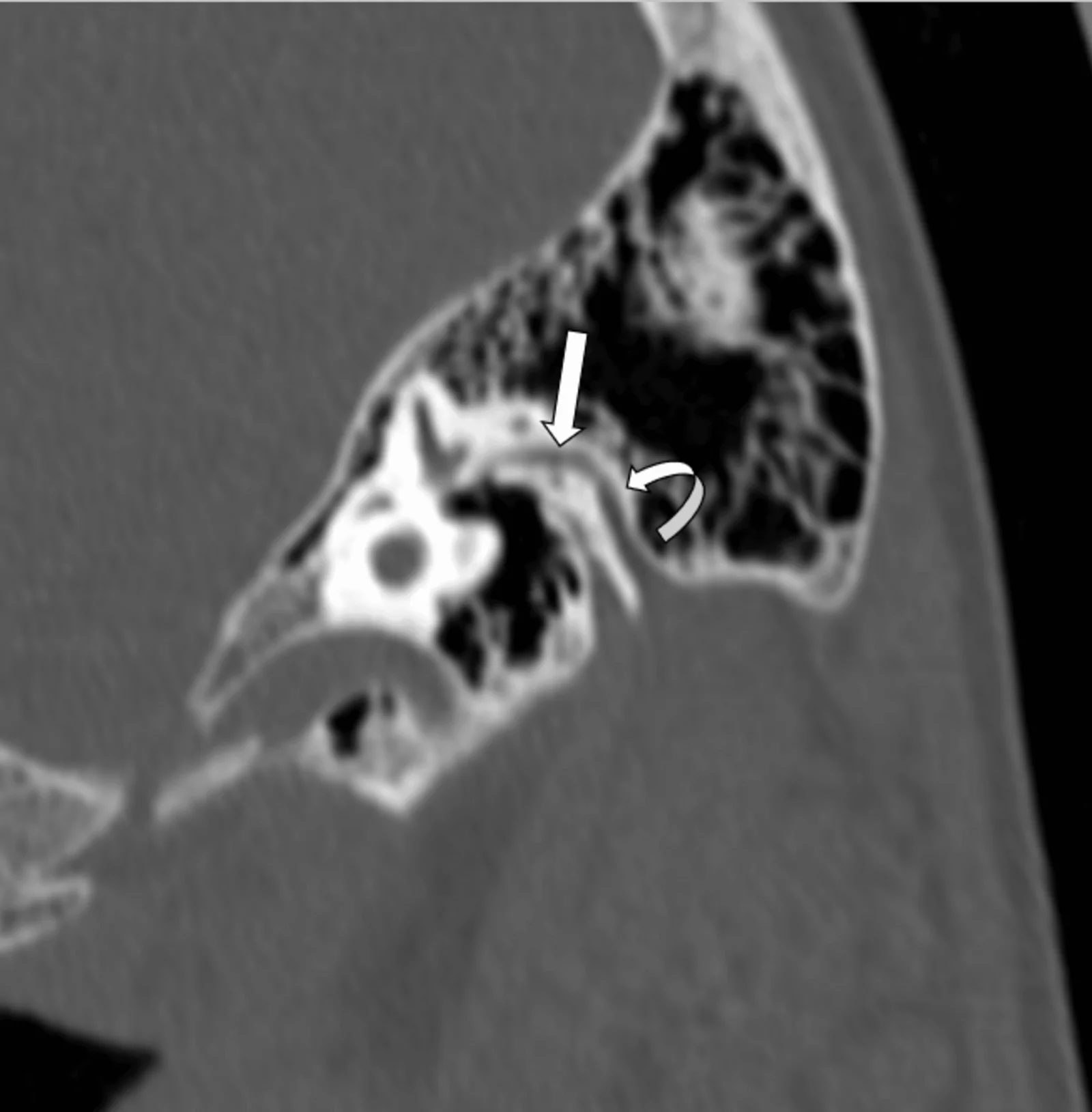
High-resolution computed tomography (HRCT) of the left temporal bone - oblique sagittal section The images demonstrate a normal, well-defined bony facial canal for comparison. Straight arrow: tympanic part of the facial nerve canal; curved arrow: mastoid part of facial nerve canal

The contralateral (left) temporal bone demonstrated a normal course and caliber of the labyrinthine, tympanic, and mastoid segments of the left facial nerve canal, along with normal inner and middle ear structures (Figures 3, 4).

Based on these radiological findings, a definitive diagnosis of isolated congenital developmental anomaly (hypoplasia/aplasia) of the right facial nerve canal was established.

## Discussion

The embryological development of the facial nerve and its surrounding bony canal is a complex, multi-stage process that makes it vulnerable to teratogenic or genetic disruption. The development of the temporal bone and otic capsule can be variable in congenital anomalies, ranging from normally formed to completely aplastic [5]. The canal is formed by a combination of periosteal ossification of the otic capsule and membranous ossification of the surrounding mesenchyme, which begins around the twentieth week of gestation and continues even after birth.

Abnormal disruption of any of the processes involved causes anomalies of both the facial nerve and other structures such as the ossicular chain and the inner ear. Because the development of the facial nerve is intimately related to the formation of its bony canal, an aplasia or hypoplasia of the nerve itself will result in a correspondingly aplastic or hypoplastic Fallopian canal. What makes the present case exceptionally rare is the strictly isolated nature of the defect. Severe hypoplasia/aplasia of the facial canal segments in the context of a normal ossicular chain, otic capsule, and middle ear pneumatization points to an extremely localized developmental arrest specific only to the facial nerve placode, sparing the adjacent structures of the first and second branchial arches [2].

HRCT is the radiological cornerstone in the evaluation of CFNP. It provides the necessary sub-millimeter spatial resolution to assess the diameter of the internal auditory canal and trace the Fallopian canal from the labyrinthine segment down to the stylomastoid foramen [4]. While HRCT brilliantly delineates the bony anatomy and definitively confirms the presence of an osseous canal defect, it cannot directly visualize the neural tissue. Magnetic resonance imaging (MRI) utilizing heavily T2-weighted, three-dimensional steady-state free precession sequences (such as CISS) is essential to directly visualize the cisternal and intracanalicular segments of the cranial nerves and definitively prove hypoplasia or aplasia of the facial nerve itself [2]. In the present case, while the absolute absence of the facial nerve cannot be proven without an MRI, the HRCT definitively confirms the severe hypoplasia and aplasia of the enclosing Fallopian canal, which serves as a highly reliable structural correlate for the clinical facial palsy.

The clinical management of developmental CFNP requires a multidisciplinary approach. Unlike traumatic CFNP, which may benefit from conservative management or surgical decompression if an evolving hematoma is suspected, developmental aplasia renders decompression futile. The primary initial focus must be on ophthalmological care to prevent exposure keratopathy due to lagophthalmos. Long-term management involves plastic and reconstructive surgery. Because the native facial musculature often atrophies or fails to develop in the absence of innervation, early radiological diagnosis is critical. It helps surgeons bypass exploratory middle ear surgeries and move directly toward facial reanimation techniques, such as cross-facial nerve grafting or free functional muscle transfers (e.g., gracilis muscle transfer), typically considered when the child is older [1].

To contextualize our findings, a comprehensive comparison of the present case with existing literature on congenital facial nerve and canal anomalies is provided in Table 1. This comparison highlights the extreme rarity of strictly isolated osseous and neural defects occurring without any accompanying external, middle, or inner ear malformations.

**Table 1:** Comparison of clinical and radiological characteristics between the present case and previous literature on congenital facial palsy HRCT: high-resolution computed tomography; MRI: magnetic resonance imaging; N/A: not applicable/not available

Author (Year) (Ref)	Study Type	Patient Age/Sex	Clinical Presentation	Imaging Modality	Key Radiologic Findings	Associated Ear Anomalies
Current Case	Case Report	4 years/Female	Right-sided facial nerve palsy since birth	HRCT	Hypoplastic labyrinthine segment; severe hypoplasia/aplasia of tympanic and mastoid segments.	None. Normal external, middle (ossicles), and inner ear structures.
Chalipat et al. (2024) [6]	Case Report	6 months/Male	Left-sided facial nerve palsy since birth	HRCT & MRI	Aplasia of the facial nerve (MRI) and facial canal (HRCT).	None. Normal inner, middle, and external ear.
Messaoud et al. (2023) [2]	Case Report	11 months/Male	Right-sided facial nerve palsy since birth	HRCT & MRI	Absence of the facial nerve on MRI; aplasia of the facial canal on HRCT.	None. Isolated defect sparing the surrounding temporal bone structures.
Baelen et al. (2023) [1]	Retrospective Cohort	Multiple (Neonates to adolescents)	Congenital facial nerve palsy	HRCT / MRI	Variable: ranging from normal scans to hypoplasia of the facial canal and nerve.	Variable. Often associated with external, middle, or inner ear malformations (syndromic).
Kumar et al. (2016) [7]	Case Report	6 months & 2 months/Males	Unilateral facial palsy since birth	MRI (3D CISS)	Complete unilateral aplasia of the facial nerve.	None. Isolated non-syndromic facial nerve aplasia.
Ciorba (2015) [3]	Review	N/A (Pediatric focus)	Facial paralysis in children	General overview	Highlights the narrowness or absence of the fallopian canal in developmental cases.	Often associated with inner ear dysplasia or branchial arch anomalies.
Jervis & Bull (2001) [8]	Case Report	7 years/N/A	Congenital facial palsy diagnosed at birth	Surgical Exploration	Complete absence of the facial nerve incidentally found during surgery.	None.
Kundu et al. (2025) [9]	Case Report	8 years/Male	Left-sided facial nerve palsy, restricted ocular abduction, tongue atrophy (Moebius spectrum).	MRI	Hypoplasia of the left facial nerve, absence of the left abducens nerve.	None (Anomaly involves multiple cranial nerves rather than ear structures).

## Conclusions

Isolated unilateral congenital hypoplasia and aplasia of the facial nerve canal, without concurrent inner or middle ear dysgenesis, is a remarkably rare clinical entity. This must be kept high on the differential diagnosis for pediatric patients presenting with facial palsy from birth, especially when there is no obstetric history of trauma. High-resolution computed tomography (HRCT) of the temporal bone is indispensable for mapping the complex osseous anatomy of the temporal bone and definitively confirms the presence of facial canal hypoplasia or aplasia. However, it should be emphasized that HRCT alone does not prove facial nerve agenesis. While MRI remains essential to directly visualize and definitively prove the absence or hypoplasia of the facial nerve itself, the demonstration of an isolated congenital hypoplastic or aplastic facial nerve canal on HRCT in a patient with congenital facial palsy strongly correlates with an underlying neural developmental defect. Recognizing this bony malformation early prevents unnecessary diagnostic exploratory surgeries and lays the groundwork for definitive, long-term facial reanimation surgery.
